# Comparison of Osteogenesis between Adipose-Derived Mesenchymal Stem Cells and Their Sheets on Poly-**ε**-Caprolactone/**β**-Tricalcium Phosphate Composite Scaffolds in Canine Bone Defects

**DOI:** 10.1155/2016/8414715

**Published:** 2016-08-16

**Authors:** Yongsun Kim, Seung Hoon Lee, Byung-jae Kang, Wan Hee Kim, Hui-suk Yun, Oh-kyeong Kweon

**Affiliations:** ^1^BK21 PLUS Program for Creative Veterinary Science Research, Research Institute for Veterinary Science and College of Veterinary Medicine, Seoul National University, Seoul 08826, Republic of Korea; ^2^College of Veterinary Medicine and Institute of Veterinary Science, Kangwon National University, Chuncheon 24341, Republic of Korea; ^3^Powder & Ceramics Division, Korea Institute of Materials Science, Changwon 51508, Republic of Korea

## Abstract

Multipotent mesenchymal stem cells (MSCs) and MSC sheets have effective potentials of bone regeneration. Composite polymer/ceramic scaffolds such as poly-*ε*-caprolactone (PCL)/*β*-tricalcium phosphate (*β*-TCP) are widely used to repair large bone defects. The present study investigated the* in vitro* osteogenic potential of canine adipose-derived MSCs (Ad-MSCs) and Ad-MSC sheets. Composite PCL/*β*-TCP scaffolds seeded with Ad-MSCs or wrapped with osteogenic Ad-MSC sheets (OCS) were also fabricated and their osteogenic potential was assessed following transplantation into critical-sized bone defects in dogs. The alkaline phosphatase (ALP) activity of osteogenic Ad-MSCs (O-MSCs) and OCS was significantly higher than that of undifferentiated Ad-MSCs (U-MSCs). The* ALP, runt-related transcription factor 2, osteopontin,* and* bone morphogenetic protein 7 *mRNA levels were upregulated in O-MSCs and OCS as compared to U-MSCs. In a segmental bone defect, the amount of newly formed bone was greater in PCL/*β*-TCP/OCS and PCL/*β*-TCP/O-MSCs/OCS than in the other groups. The OCS exhibit strong osteogenic capacity, and OCS combined with a PCL/*β*-TCP composite scaffold stimulated new bone formation in a critical-sized bone defect. These results suggest that the PCL/*β*-TCP/OCS composite has potential clinical applications in bone regeneration and can be used as an alternative treatment modality in bone tissue engineering.

## 1. Introduction

Synthetic bone substitutes such as collagen, hydroxyapatite (HA), *β*-tricalcium phosphate (*β*-TCP), and synthetic polymers are currently available for bone tissue regeneration. Ceramic scaffolds that consist of HA and *β*-TCP have been widely used to repair bone defects in clinical applications, since they have good biocompatibility and a microstructure similar to the mineral component of natural bone [1, 2]. Poly-*ε*-caprolactone (PCL), a type of polymer-based composite, has also been used for bone tissue engineering owing to its biodegradability, biocompatibility, and low inflammatory response [3, 4]. Some recent studies have examined the feasibility of using composite polymer/ceramic scaffolds such as PCL/*β*-TCP so as to combine the advantages of each material [4–6].

Cell-based tissue engineering is a promising alternative approach to bone regeneration. In particular, mesenchymal stem cells (MSCs) show great potential for therapeutic use in bone tissue engineering due to their capacity for osteogenic differentiation and regeneration [7]. However, transplanted single-cell suspensions do not attach, survive, or proliferate on target tissues [8]. To overcome this limitation, cell sheet technology has been developed to enhance the regenerative capacity of tissue-engineered products [9, 10]. Cell sheets are beneficial for cell transplantation because they preserve cell-cell junctions as well as endogenous extracellular matrix (ECM), thereby ensuring homeostasis of the cellular microenvironment for the delivery of growth factors and cytokines that promote tissue repair over a prolonged period of time.

We hypothesized that combining polymer and ceramic scaffolds and MSCs or MSC sheets could accelerate and enhance bone regeneration in large bone defects. In this study, canine adipose-derived MSC (Ad-MSC) sheets were generated by cell sheet technology, and the osteogenic potential of Ad-MSCs and Ad-MSC sheets was investigated* in vitro*. In addition, composite PCL/*β*-TCP scaffolds seeded with Ad-MSCs or wrapped with osteogenic cell sheets were constructed and assessed for their osteogenic potential after transplantation into critical-sized bone defects in dogs.

## 2. Materials and Methods

### 2.1. Isolation and Culture of Canine Ad-MSCs

The study protocol was approved by the Institutional Animal Care and Use Committee of Seoul National University (SNU-140801-1). MSCs derived from canine hip adipose tissue were isolated and characterized [11]. The tissue was collected aseptically from the subcutaneous fat of a 2-year-old beagle dog under anesthesia, washed with Dulbecco's phosphate-buffered saline (DPBS; Thermo Fisher Scientific Inc., USA), minced, and then digested with collagenase type I (1 mg/mL; Sigma-Aldrich) at 37°C for 30–60 min with intermittent shaking. The suspension was filtered through a 100 *μ*m nylon mesh and centrifuged to separate floating adipocytes from stromal cells. Preadipocytes in the stromal vascular fraction were plated at 8,000–10,000 cells/cm^2^ in T175 culture flasks containing Dulbecco's modified Eagle's medium (Thermo Fisher Scientific Inc., USA) supplemented with 3.7 g/L sodium bicarbonate, 1% penicillin and streptomycin, 1.7 mM l-glutamine, and 10% fetal bovine serum (Thermo Fisher Scientific Inc., USA). Cells were incubated in a humidified atmosphere at 37°C and 5% CO_2_. Unattached cells and residual nonadherent red blood cells were removed after 24 h by washing with PBS, and the culture medium was replaced every 2 days. Cells were used for experiments after the third passage.

### 2.2. Preparation of Osteogenic Cell Sheet (OCS) and Ad-MSC Cultures

OCS was prepared as previously described [9]. Briefly, Ad-MSCs were seeded at a density of 1 × 10^4^ cells/cm^2^ in a 100 mm culture dish and cultured in growth medium containing 0.1 *μ*M dexamethasone (Sigma-Aldrich, USA) and 82 *μ*g/mL l-ascorbic acid 2-phosphate (A2-P, Sigma-Aldrich, USA) for 10 days. As a positive control for Ad-MSCs induced to undergo osteogenic differentiation (O-MSCs), cells were seeded at the same density and cultured in growth medium containing 0.1 *μ*M dexamethasone, 15 *μ*g/mL A2-P, and 10 mM *β*-glycerophosphate (Sigma-Aldrich, USA) [12, 13]. Undifferentiated Ad-MSCs (U-MSCs, negative control) were cultured in unsupplemented growth medium for 10 days. Morphological changes in cells during culture were monitored under an inverted light microscope (Olympus Corp., Japan). The OCS was fixed in 4% paraformaldehyde, embedded in paraffin, sectioned at a thickness of 4 *μ*m, and stained with hematoxylin and eosin (H&E).

### 2.3. Alkaline Phosphatase (ALP) Activity Measurement

Cells cultured in 100 mm dishes were used for measurement of ALP activity using an ALP assay kit (Takara Bio Inc., Japan) according to the manufacturer's instructions. Briefly, p-nitrophenyl phosphate (pNPP) solution was prepared by dissolving 24 mg pNPP substrate in 5 mL ALP buffer. Cells were scraped into 200 *μ*L extraction solution, homogenized, and sonicated. The cleared supernatant was collected after centrifugation at 13,000 ×g and 4°C for 10 min. A 50 *μ*L volume of cell lysate supernatant was mixed with 50 *μ*L pNPP substrate solution and incubated at 37°C for 30 min. After adding 50 *μ*L stop solution (0.5 N NaOH), absorbance was measured at 405 nm with a spectrophotometer.

### 2.4. Quantification of Mineralization

Alizarin Red S (ARS) staining was used to detect calcium mineralization. Cells cultured in 100 mm dishes for 10 days were washed twice with DPBS and fixed with 4% paraformaldehyde (Wako, Japan) at room temperature for 10 min. Cells were then washed three times with distilled water, and 3 mL of 40 mM ARS (Sigma-Aldrich, USA, pH 4.1–4.3) was added to each dish, followed by incubation at room temperature for 20 min with gentle shaking. Excess dye was removed by aspiration and cells were washed three times with distilled water. For quantification of staining, the ARS was solubilized in 2 mL cetylpyridinium chloride (Sigma-Aldrich, USA) for 1 h [14], and the absorbance at 570 nm was measured with a spectrophotometer.

### 2.5. Gene Expression Analysis

Total RNA was isolated from cells using the Hybrid-RTM RNA extraction kit (GeneAll Bio, Korea) according to the manufacturer's protocol. RNA concentration was determined by measuring optical density at 260 nm with a NanoDrop ND-1000 spectrophotometer (NanoDrop Technologies, USA). cDNA was synthesized from RNA using a commercially available cDNA synthesis kit (Takara Bio, Japan). Quantitative reverse transcription polymerase chain reaction (qRT-PCR) was carried out on an ABI 7300 Real-Time PCR system (Applied Biosystems, USA) and SYBR Premix Ex Taq (Takara Bio, Japan). Primer sequences are listed in Table 1. Expression levels of target genes were normalized to the level of glyceraldehyde 3-phosphate dehydrogenase (GAPDH) and quantitated with the ΔΔCt method [15].

### 2.6. Fabrication of PCL/*β*-TCP Scaffolds

**Figure 1:**
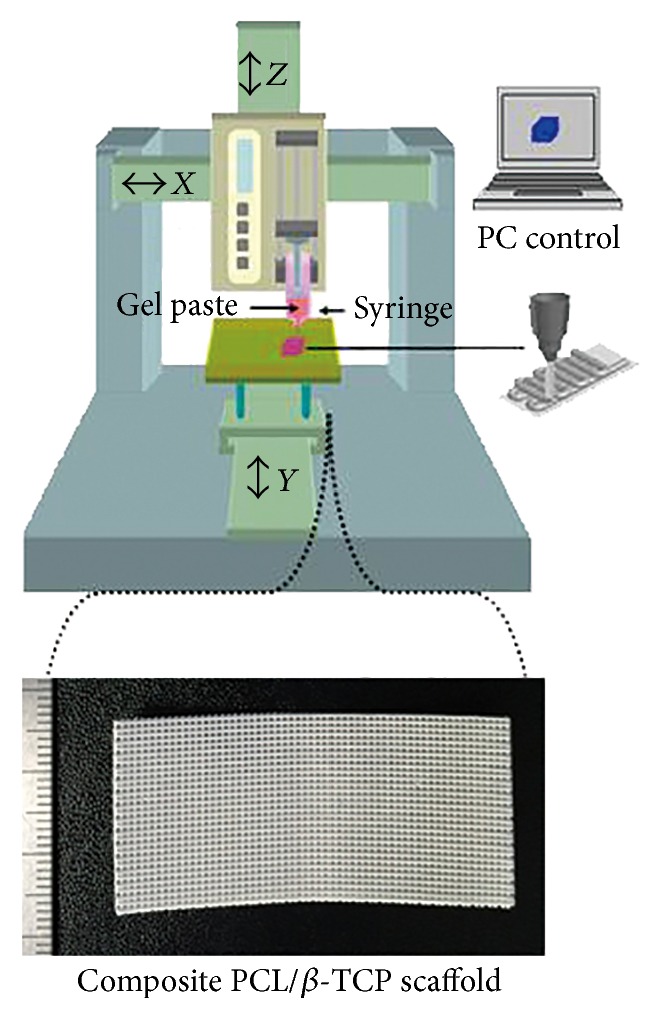
Photograph of a fabricated composite PCL/*β*-TCP scaffold. Sheet-type porous scaffolds (50 × 25 mm, five layers) were constructed by extruding the gel paste onto a substrate using a three-dimensional printing system.

PCL was dissolved in chloroform at 40°C. NaCl and *β*-TCP were ground and sieved, and granules between 25 and 33 *μ*m were selected. *β*-TCP was prepared by calcination of nano-TCP (Merck, USA) at 1,000°C for 4 h. Selected NaCl granules were mixed with predetermined amounts of ceramic particles (1 : 1 = NaCl : PCL, 1.5 : 1 = ceramic : PCL, weight ratios). Combined powders were mixed with the PCL suspension to produce a homogeneous paste. Sheet-type porous scaffolds (50 × 25 mm, five layers) were constructed by extruding the gel paste onto a substrate using a three-dimensional (3D) printing system (Figure 1). The shapes and sizes of the PCL/*β*-TCP scaffold were designed using a computer system. NaCl was removed by immersing the scaffold in deionized water to produce macrosized pores in strut and the water was replaced every 2 h with fresh water at 30°C after sufficient drying of the scaffold.

### 2.7. Preparation of Scaffold with Ad-MSCs and Cell Sheet

Scaffolds were immersed in DPBS for 24 h. Ad-MSCs (~1 × 10^6^) were seeded on the scaffolds in a 100 mm dish for the PCL/*β*-TCP/U-MSCs group. After 24 h of incubation, the medium was replaced with osteoinductive medium for the PCL/*β*-TCP/O-MSCs group. The culture was maintained for 10 days at 37°C and 5% CO_2_, and the medium was changed every 48 h. For the PCL/*β*-TCP/OCS group, the scaffold was wrapped with four OCS after 10 days of culture. Cell-free scaffolds cultured in growth medium under the same conditions were used as controls.

### 2.8. Animal Experiments

Beagle dogs (*n* = 20, 2-3-year-old) weighing 8.7 ± 1.4 kg were used in the study. Dogs were handled in accordance with the animal care guidelines of the Institute of Laboratory Animal Resources, Seoul National University, Korea. The dogs were assigned to one of five groups (*n* = 4 in each): PCL/*β*-TCP (control), PCL/*β*-TCP/U-MSCs, PCL/*β*-TCP/O-MSCs, PCL/*β*-TCP/OCS, and PCL/*β*-TCP/O-MSCs/OCS. The Institutional Animal Care and Use Committee of Seoul National University approved the experimental design. Dogs were medicated and anesthetized with tramadol (4 mg/kg by intravenous (i.v.) injection; Toranzin; Samsung Pharmaceutical Co., Korea), propofol (6 mg/kg i.v.; Provive; Claris Lifesciences, Indonesia), and atropine sulfate (0.05 mg/kg by subcutaneous injection; Jeil Pharmaceutical Co., Korea). Anesthesia was maintained with isoflurane (Forane solution, Choongwae Pharmaceutical Co., Korea) at 1.5 minimum alveolar concentration throughout the procedure. Electrocardiography, pulse oximetry, respiratory gas analysis, and rectal temperature measurement were carried out using an anesthetic monitoring system (Datex-Ohmeda S/5; GE Healthcare, UK). Under sterile conditions, a craniomedial incision was made to the skin to expose the diaphysis of the left radius. To create a critical-sized segmental defect in the radial diaphysis, a 15 mm long segmental defect was made to the middle portion of the diaphysis using an oscillating saw (Stryker, USA) as previously described [16, 17]. Overlying periosteum was also resected from the defect area. Defects were surrounded by the experimental scaffold. A nine-hole, 2.7 mm dynamic compression plate (DePuy Synthes, Switzerland) was placed on the cranial aspect of the radius. The soft tissue was closed with 3-0 polydioxanone sutures (Ethicon, USA), and the skin was closed with 4-0 nylon sutures. All the animals were bandaged for 2 days after operation.

### 2.9. Microcomputed Tomography (CT) Bone Imaging

Dogs were sacrificed 12 weeks after implantation. The radius segment was excised, trimmed, and fixed in 10% formaldehyde. Samples were scanned using a micro-CT system (Skyscan; Bruker Corp., Belgium) and 3D images were reconstructed; the volume of newly formed bone within bone defects was calculated using the auxiliary software (Bruker Corp., Belgium).

### 2.10. Histological Analysis

After micro-CT measurement, specimens were decalcified in 10% ethylenediaminetetraacetic acid for 4 weeks at room temperature and then dehydrated through a graded series of alcohol, embedded in paraffin, sectioned at a thickness of 5 or 8 *μ*m, and stained with H&E or Masson's trichrome according to standard protocols.

### 2.11. Statistical Analysis

Results are expressed as mean ± standard deviation. Statistical analysis was performed using SPSS v.21.0 software (IBM Corp., USA). Group means were compared with the Kruskal-Wallis tests followed by Mann-Whitney *U* tests. A *P* value of less than 0.05 was considered statistically significant.

## 3. Results

### 3.1. Cell Sheet Formation and Osteogenic Differentiation

**Figure 2:**
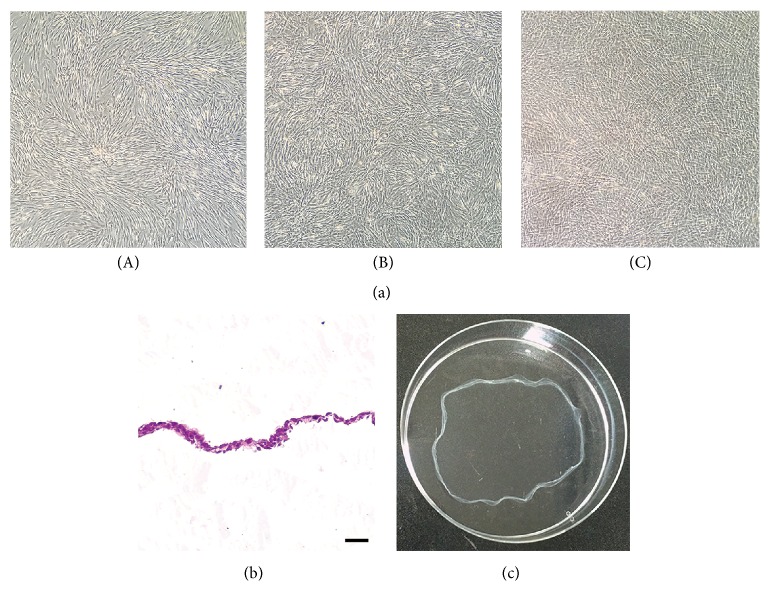
Morphological characteristics of the adipose-derived mesenchymal stem cells (Ad-MSCs) and Ad-MSC sheets. (a) (A) undifferentiated Ad-MSCs, (B) osteogenic Ad-MSCs, and (C) osteogenic Ad-MSC sheets observed under a phase contrast microscope. (b) OCS was composed of multiple layers of cells surrounded by ECM. (c) OCS was easily detached by cell scraper. Scale bars = 25 *μ*m.

**Figure 3:**
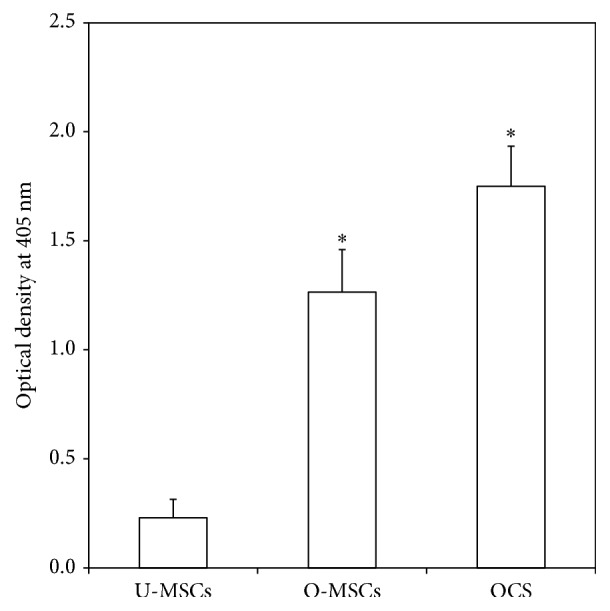
Quantification of alkaline phosphatase (ALP) activity. ALP activity was significantly higher in the O-MSCs and OCS than in the U-MSCs group (^*∗*^ *P* < 0.05).

**Figure 4:**
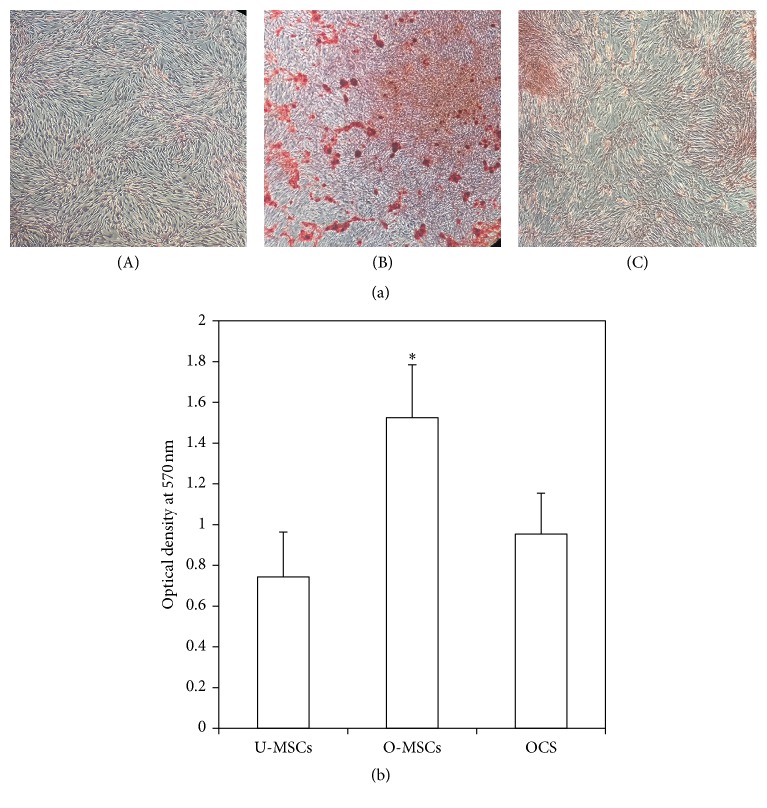
Alizarin Red S (ARS) staining. (a) (A) U-MSCs, (B) O-MSCs, and (C) OCS were stained using ARS solution. Calcium-rich granules were clearly visible in the O-MSCs group. (b) The degree of mineralization was greater in the O-MSCs group (^*∗*^ *P* < 0.05).

U-MSCs and O-MSCs cultured for 10 days exhibited a spindle-shaped, fibroblast-like morphology with clearly delineated cell margins (Figures 2(a)(A) and 2(a)(B)). However, OCS appeared to overlap and were stacked on top of one another, with indistinguishable cell-cell boundaries (Figure 2(a)(C)). The OCS was composed of two to four layers of cells surrounded by ECM (Figure 2(b)), and it was easily detached by cell scraper (Figure 2(c)). ALP activity was higher in the O-MSCs and OCS than in the U-MSCs group (*P* < 0.05; Figure 3). After staining with ARS, calcium-rich granules were clearly visible in the O-MSCs group (Figure 4(a)(B)), whereas no nodules were observed in the U-MSCs and OCS groups (Figures 4(a)(A) and 4(a)(C)). The degree of ARS staining was also greater in the O-MSCs group (Figure 4(b)).

### 3.2. Expression of Osteogenic Differentiation Markers in Ad-MSCs and Matrix Cell Sheets

**Figure 5:**
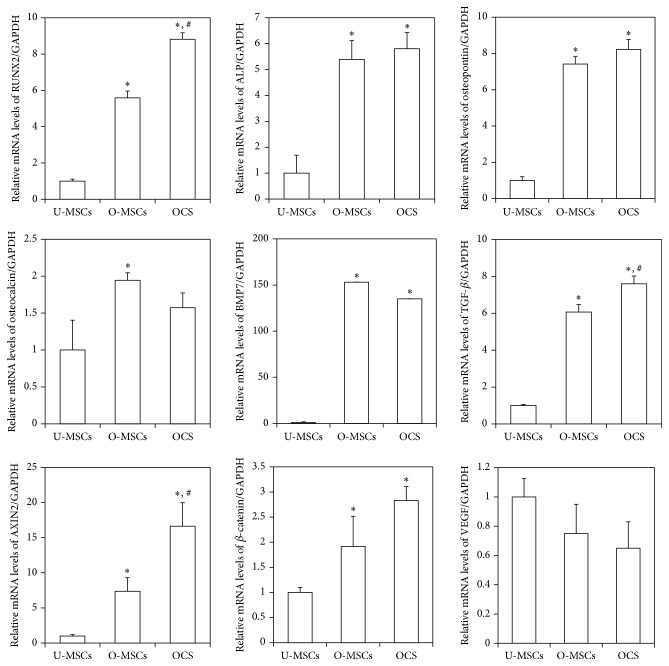
Expression of osteogenic differentiation markers. The expression of* RUNX2, ALP, osteopontin, BMP7*, and* TGF-β* mRNA was significantly upregulated in O-MSCs and OCS (^*∗*^ *P* < 0.05).* RUNX2* and* TGF-β* transcript levels were higher in OCS than in the O-MSCs group (^#^ *P* < 0.05).* AXIN2* and *β-catenin* mRNA expression was upregulated in O-MSCs and OCS (^*∗*^ *P* < 0.05). *∗*: compared to the U-MSCs group, #: compared to the O-MSCs group.

The expression of* runt-related transcription factor 2 (RUNX2), ALP, osteopontin, bone morphogenetic protein 7 (BMP7),* and* transforming growth factor beta (TGF-β*) mRNA was significantly upregulated in O-MSCs and OCS compared to the U-MSCs control (*P* < 0.05; Figure 5).* RUNX2* and* TGF-β* transcript levels were higher in OCS than in the O-MSCs group (*P* < 0.05). The involvement of the Wnt/*β*-catenin signaling pathway was investigated by evaluating* axis inhibition protein 2 (AXIN2)* and *β-catenin* expression. Both transcripts were upregulated in O-MSCs and OCS relative to U-MSCs (*P* < 0.05). The mRNA level of* vascular endothelial growth factor (VEGF)* tends to be downregulated in O-MSCs and OCS as compared to the U-MSCs group.

### 3.3. *In Vivo* Bone Regeneration in Canine Radial Defects

**Figure 6:**
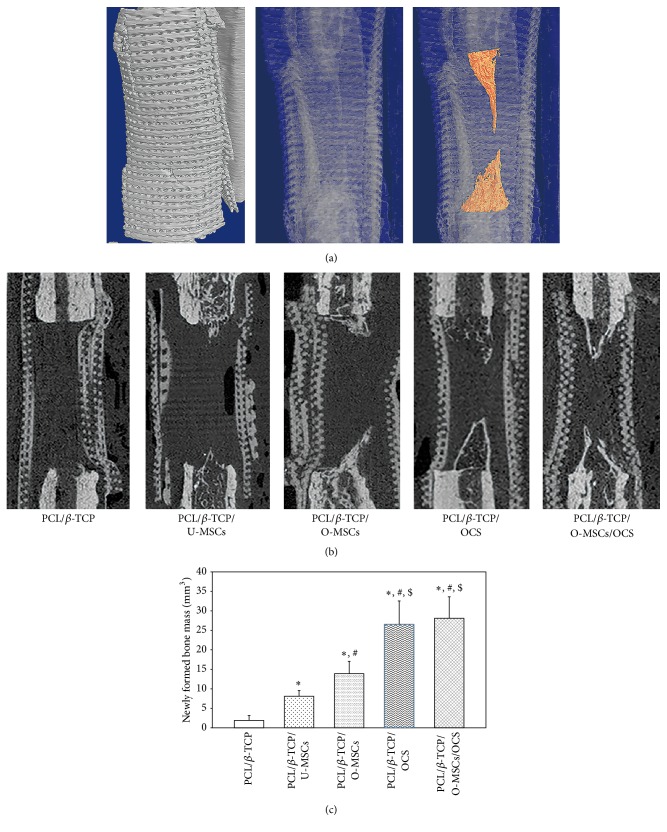
Bone regeneration in canine radial defects. (a) 3D reconstructed image and (b) sagittal view image showed that new bone formation was detected within defects at the bone margin. (c) Quantitative 3D micro-CT analysis revealed that groups with cell sheets (with or without O-MSCs) showed a greater volume of newly formed bone than the other groups (^*∗*, #, $^ *P* < 0.05). *∗*: compared to the PCL/*β*-TCP group, #: compared to the PCL/*β*-TCP/U-MSCs group, and $: compared to the PCL/*β*-TCP/O-MSCs group.

New bone was detected within defects at the bone margin. In the 3D reconstructed image, the cone-shaped newly formed bone was visible (Figure 6(a)). From the sagittal view, the bone volume was discernible (Figure 6(b)), and a quantitative 3D micro-CT analysis revealed the following values for newly formed bone mass: PCL/*β*-TCP, 1.89 ± 1.27 cm^3^; PCL/*β*-TCP/U-MSC, 8.10 ± 1.46 cm^3^; PCL/*β*-TCP/O-MSC, 16.81 ± 3.15 cm^3^; PCL/*β*-TCP/OCS, 26.53 ± 6.02 cm^3^; and PCL/*β*-TCP/O-MSC/OCS, 28.11 ± 5.5 cm^3^ (Figure 6(c)). The amount of new bone formed was greater in all experimental groups than in the PCL/*β*-TCP group (*P* < 0.05). Moreover, groups with cell sheets (with or without O-MSCs) showed a greater volume of newly formed bone than the other groups (*P* < 0.05).

### 3.4. Histological Evaluation

**Figure 7:**
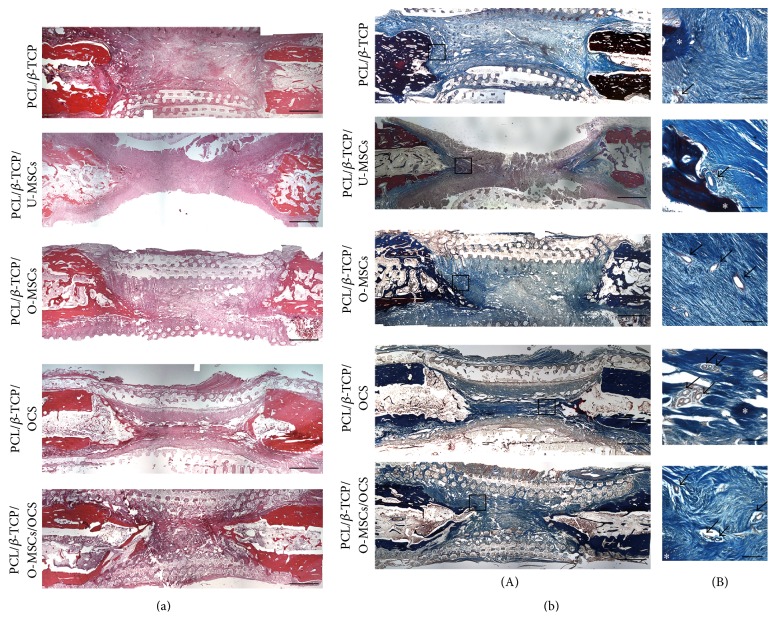
Histological analysis. (a) In hematoxylin and eosin staining, most of the defect areas were filled with fibrous connective tissue, and newly formed bone tissue had a woven, trabecular appearance. (b) Masson's trichrome staining revealed abundant collagenous tissue around the regenerated tissue. Vasculatures were observed inside and around the new bone. Asterisks and arrows indicate bone tissue and vasculatures, respectively. Scale bars = ((a), (b)(A)) 200*μ*m, ((b)(B)) 15*μ*m.

At 12 weeks after implantation, decalcified paraffin sections were stained with H&E and Masson's trichrome to identify regenerated bone in defect areas. In all experimental groups, new bone was observed in longitudinal sections throughout the segmental defect and there was no obvious inflammation. Most of the defect areas were filled with fibrous connective tissue and newly formed bone tissue had a woven, trabecular appearance (Figure 7(a)). Masson's trichrome staining revealed abundant collagenous tissue around the regenerated tissue (Figure 7(b)(A)). In addition, vasculatures were observed inside and around the new bone (Figure 7(b)(B)).

## 4. Discussion

The present study investigated the osteogenic potential of Ad-MSCs and Ad-MSC sheets, as well as that of composite PCL/*β*-TCP scaffolds seeded with Ad-MSCs or wrapped with OCS after their transplantation into critical-sized bone defects in dogs. MSCs have been reported to promote fracture repair; however, injection of single-cell suspensions leads to uneven distribution and weak adhesion of cells, which may ultimately result in cell death [8]. Additionally, the transplantation of isolated cells is impractical for bone regeneration in large-sized defects, which would require an adequate supply of cells. This is provided by cell sheets, which have intact cell-cell junctions and ECM that confer mechanical support and thereby maintain the integrity of the transplant [18]. In this study, we create a cell sheet using A2-P; the OCS had multiple layers of proliferating cells with ECM formation. A2-P is a stable form of ascorbic acid that plays a role in collagen biosynthesis and ECM deposition [19]. The OCS was readily detached from the culture dish using a scraper rather than a proteolytic agent such as trypsin, which preserved critical cell surface proteins such as ion channels, growth factor receptors, and cell-to-cell junction proteins.

MSCs are capable of producing multiple mesenchymal cell lineages under specific culture conditions [7, 12]. Differentiation into the osteoblastic lineage is induced by culturing in osteoinductive medium containing dexamethasone, vitamin C, and *β*-glycerophosphate. In this study, the O-MSCs and OCS showed strong osteogenic potential, as evidenced by upregulation of the osteogenic differentiation markers such as* RUNX2, ALP, *and* osteopontin *as well as the increase in ALP activity relative to undifferentiated Ad-MSCs. These osteogenic effects of O-MSCs and OCS correspond well with those previously reported [20, 21]. In our* in vivo* study, the PCL/*β*-TCP/O-MSC group showed more extensive bone regeneration than the PCL/*β*-TCP/U-MSC group, likely due to the higher osteogenic potential of O-MSCs relative to U-MSCs. Moreover, there was more newly formed bone in the PCL/*β*-TCP/OCS and PCL/*β*-TCP/O-MSC/OCS groups than in those without OCS. The enhanced bone formation might be due to the delivery of osteogenic cells and ECM to the defect sites by MSC sheets.

As for the role of MSCs in bone tissue engineering, besides osteogenic differentiation, MSCs are thought to exert therapeutic effects via secretion of trophic factors that provide a supportive microenvironment for cell survival, renewal, and differentiation [22]. It has been suggested that wrapped cell sheets function as a tissue-engineered periosteum at bone defect sites. A biomimetic periosteum can maintain homeostasis of the cellular microenvironment by delivering growth factors. A previous study showed that paracrine factors of MSCs play a positive role in bone repair [23, 24]. During bone healing, the proliferation and osteoblastic differentiation of endogenous or exogenous MSCs are influenced by various growth factors, among which TGF-*β* and BMPs play a major role. Both are members of the TGF-*β* superfamily, a group of dimeric proteins, acting as growth and differentiation factors. The BMP/TGF-*β* signaling induces MSCs differentiation into osteoblast via activation of intracellular pathways such as SMAD and mitogen-activated protein kinase signaling [25, 26]. Wnt signaling is also crucial in bone regeneration. Wnt/*β*-catenin signaling pathway promotes osteoblastogenesis, activation of osteoblast activity, inhibition of osteoclast activity, and increase in bone mass [1, 27]. In the present study, OCS showed higher expression of* RUNX2*,* BMP7, TGF-β, AXIN2, *and *β-catenin*, suggesting that the induction of bone regeneration by OCS occurs via activation of the BMP/TGF-*β* and Wnt signal pathways.

Osteogenesis requires a well-developed vascular supply. It has been proposed that MSCs and cell sheets stimulate bone formation by inducing vascularization [7, 9, 21]. Neovascularization helps to overcome the hypoxic environment and facilitate bone formation. VEGF promotes angiogenesis and indirectly stimulates bone formation by inducing the ingress of osteoprogenitor cells. In the present study, U-MSCs, O-MSCs, and OCS expressed* VEGF*, which corresponded to the formation of a vascular network around newly formed bone tissue following transplantation of scaffolds into bone defects. This neovascularization may also have positive effects on the bone tissue regeneration.

In this study, we used a PCL/*β*-TCP composite as a scaffold for bone regeneration. PCL is a biodegradable polymer with a porous 3D structure [28]. This scaffold has approximately 500 *µ*m sized pores and 70% of porosity; thus, it has large surface area. Ceramic powders such as *β*-TCP, which is an inorganic component of bone, may enhance the mechanical properties of the PCL scaffolds. Recent studies have shown that *β*-TCP has good osteoconductivity and biocompatibility and promotes MSCs adherence, survival, and osteogenic differentiation [29, 30]. Thus, in large bone defects, the PCL/*β*-TCP composite may provide structural and mechanical support and enhance interactions between scaffold and cells or cell sheets in a manner that is conducive to bone regeneration.

## 5. Conclusion

Our results demonstrate that osteogenic Ad-MSC sheets have strong osteogenic potential. Moreover, OCS combined with a PCL/*β*-TCP composite scaffold stimulated new bone formation to repair critical-sized bone defects in dogs. Ad-MSC sheets not only deliver osteogenic cells along with ECM, but also secrete trophic factors at defect sites for the bone regeneration. Our findings indicate that the PCL/*β*-TCP/OCS composite has a therapeutic potential for the treatment of bone defects and could be used to enhance current treatment practices.

## Figures and Tables

**Table 1:** Primers sequences used for quantitative reverse transcription PCR.

Target gene		Primer sequence (5′-3′)
RUNX2	Forward	TGTCATGGCGGGTAACGAT
	Reverse	TCCGGCCCACAAATCTCA

ALP	Forward	TCCGAGATGGTGGAAATAGC
	Reverse	GGGCCAGACCAAAGATAGAG

Osteopontin	Forward	GATGATGGAGACGATGTGGATA
	Reverse	TGGAATGTCAGTGGGAAAATC

Osteocalcin	Forward	CTGGTCCAGCAGATGCAAAG
	Reverse	GGTCAGCCAGCTCGTCACAGTT

BMP7	Forward	TCGTGGAGCATGACAAAGAG
	Reverse	GCTCCCGAATGTAGTCCTTG

AXIN	Forward	ACGGATTCAGGCAGATGAAC
	Reverse	CTCAGTCTGTGCCTGGTCAA

*β*-catenin	Forward	TACTGAGCCTGCCATCTGTG
	Reverse	ACGCAGAGGTGCATGATTTG

VEGF	Forward	CTATGGCAGGAGGAGAGCAC
	Reverse	GCTGCAGGAAACTCATCTCC

GAPDH	Forward	CATTGCCCTCAATGACCACT
	Reverse	TCCTTGGAGGCCATGTAGAC
